# Tomato Apical Leaf Curl Virus: A Novel, Monopartite Geminivirus Detected in Tomatoes in Argentina

**DOI:** 10.3389/fmicb.2017.02665

**Published:** 2018-01-12

**Authors:** Carlos G. Vaghi Medina, Elin Teppa, Verónica A. Bornancini, Ceferino R. Flores, Cristina Marino-Buslje, Paola M. López Lambertini

**Affiliations:** ^1^Area de Interacción Planta-Patógeno-Vector, Instituto de Patología Vegetal, Centro de Investigaciónes Agropecuarias, Instituto Nacional de Tecnología Agropecuaria, Córdoba, Argentina; ^2^Instituto de Investigaciones Bioquímicas de Buenos Aires, Fundación Instituto Leloir, Buenos Aires, Argentina; ^3^Estación Experimental Agropecuaria Yuto, Instituto Nacional de Tecnología Agropecuaria, Yuto, Argentina

**Keywords:** ToALCV, coat protein, treehopper, recombination, specificity-determining positions, sequence similarity network, tomato, Argentina

## Abstract

Plant viruses that are members of the *Geminiviridae* family have circular single-stranded DNA (ssDNA) genome and are responsible for major crop diseases worldwide. We have identified and characterized a novel monopartite geminivirus infecting tomato in Argentina. The full-length genome was cloned and sequenced. The genome-wide pairwise identity calculation that resulted in a maximum of 63% identity with all of other known geminiviruses indicated that it is a new geminivirus species. Biolistic infected plants presented interveinal yellowing, apical leaf curling and extreme root hypotrophy. Thus, the name proposed for this species is tomato apical leaf curl virus (ToALCV). The phylogenetic inferences suggested different evolutionary relationships for the replication-associated protein (Rep) and the coat protein (CP). Besides, the sequence similarity network (SSN) protein analyses showed that the complementary-sense gene products (RepA, Rep and C3) are similar to capulavirus while the viron-sense gene products (CP, MP and V3) are similar to topocuvirus, curtovirus and becurtovirus. Based on the data presented, ToALCV genome appears to have “modular organization” supported by its recombination origin. Analyses of the specificity-determining positions (SDPs) of the CP of geminiviruses defined nine subgroups that include geminiviruses that share the same type of insect vector. Our sequences were clustered with the sequences of topocuvirus, whose vector is the treehopper, *Micrutalis malleifera*. Also, a set of the highest scored amino acid residues was predicted for the CP, which could determine differences in virus transmission specificity. We predict that a treehopper could be the vector of ToALCV, but transmission assays need to be performed to confirm this. Given everything we demonstrate in this paper, ToALCV can be considered a type member of a new putative genus of the *Geminiviridae* family.

## Introduction

Argentina is South America’s third largest producer of fresh tomato after Brazil and Chile (Food and Agriculture Organization [FAO], 2014). Plant viruses that are members of the *Geminiviridae* family are responsible for major crop diseases worldwide (Varma and Malathi, 2003; Scholthof et al., 2011; Rybicki, 2015). Their circular ssDNA genome varies between 2.5 and 5.2 kb in length and is packed into twinned icosahedral particles (Zhang et al., 2001). Nowadays, this family comprises nine genera: *Becurtovirus, Begomovirus, Capulavirus, Curtovirus, Eragrovirus, Grablovirus, Mastrevirus, Topocuvirus*, and *Turncurtovirus*, classified according to the differences in their genome organization, their genome-wide pairwise sequence identities, their insect vector and host range. Known insect vectors are: whiteflies in the case of *Begomovirus*; leafhoppers for *Becurtovirus, Curtovirus, Turncurtovirus* and *Mastrevirus;* treehoppers for *Topocuvirus* and *Grablovirus*, and finally, aphids in the case of *Capulavirus* (Zerbini et al., 2017). *Capulavirus* and *Grablovirus* were the two last genera to be established and the most divergent viruses within the *Geminiviridae* family (Varsani et al., 2017). *Capulaviruses* include the species: *Euphorbia caput medusae latent virus* (EcmLV), *French bean severe leaf curl virus* (FbSLCV), *Alfalfa leaf curl virus* (ALCV) and *Plantago lanceolata latent virus* (PILV) (Bernardo et al., 2013, 2016; Roumagnac et al., 2015; Susi et al., 2017). ALCV was the only member that could be observed by electron microscopy and it is transmitted by the *Aphis cracivora* (Roumagnac et al., 2015). For the moment, there is only one Grablovirus species, *Grapevine red blotch virus*, and its proposed vector is the species *Spissistilus festinus* (GRBV) (Krenz et al., 2012; Poojari et al., 2013). In addition, there are other characterized divergent geminiviruses which are not yet assigned to a genus, like the citrus chlorotic dwarf-associated virus (CCDaV), the mulberry mosaic dwarf-associated virus (MMDaV), the apple geminivirus (AGV) and the grapevine geminivirus A (Loconsole et al., 2012; Liang et al., 2015; Ma et al., 2015; Al Rwahnih et al., 2017). All these viruses are putative members of the *Geminiviridae* family since they have a ssDNA genome, shared conserved protein domains and common replication motifs in their intergenic regions, like the hairpin-loop, with the characteristic geminivirus nonanucleotide (TAATATTAC).

Geminivirus-related tomato diseases in America are common, but generally, they are etiologically related with begomovirus and topocuvirus (Stanley et al., 1986; Briddon et al., 1996; Andrade et al., 2006; Castillo-Urquiza et al., 2008; Albuquerque et al., 2012; Vaghi Medina and López Lambertini, 2012; Melgarejo et al., 2013; Paz-Carrasco et al., 2014; Vaghi Medina et al., 2015). Here we identify, molecular characterize and demonstrate the infectivity of a new highly divergent species isolated from *Solanum lycopersicum* plants in Argentina. In addition, phylogenetic and recombination analyses were carried out. We use sequence similarity network (SSN) analysis to study the relationships of sequences and function among proteins codified by the different genus of the *Geminiviridae* family. Moreover, the specificity-determining positions (SDPs) of the coat protein (CP) analyses allow us to predict the putative vector of this virus. The name tomato apical leaf curl virus (ToALCV) is proposed and could be considered a type member of a new putative genus of the *Geminiviridae* family.

## Materials and Methods

### Plant Material and Molecular Characterization

Symptomatic tomato plants were collected in Yuto, Jujuy province, Argentina. Total DNA was purified with the Nucleospin^®^ Plant II total DNA purification kit (Macherey-Nagel). Genomic DNA was amplified by rolling circle amplification (RCA) with phi29 polymerase (Templi-phi^TM^, GE Healthcare). RCA products were digested with *Pst*I to obtain a complete monomeric genomic component, which was cloned into a pBluescriptII SK+, digested with *Pst*I and dephosphorylated. The virus genome was sequenced by Primer Walking in Macrogen Inc. (Korea). Open reading frames (ORFs) were identified with the ORF finder in the Geneious R9 software^1^ (Kearse et al., 2012). BLASTX, BLASTN and BLASTP algorithms^2^ were used to identify which of the sequences were most related. After identifying the closest sequences with the BLAST algorithm, we used Muscle to align them with the query and calculate the pairwise identity without considering the gaps. Known protein domains were identified in the translated putative ORFs with SMART online software (Schultz et al., 1998; Letunic et al., 2015), the NCBI Conserved Domain tool (Marchler-Bauer et al., 2015) and the InterProScan tool in Geneious R9.

### Sequence Similarity Network Analysis

We performed a SSN analysis to visualize the relationships of sequences and function among proteins from different genus of the *Geminiviridae* family. We retrieved sequences from the protein NCBI database, corresponding to the complementary-sense genes (*C1*, C2, *C3*, *C4*, and *C5*) of the 9 genera. As a large number of sequences were retrieved for mastrevirus and begomovirus (1785 and 18870, respectively), sequences of those groups were clustered at 95% of identity using the CD-hit (Li and Godzik, 2006). We also filtered out sequences with less than 50 residues in length, after which 2910 sequences remained, which belonged to the 5 proteins that were used to create a custom BLAST database. The pairwise relationships between sequences were calculated by an all-against-all BLAST in the custom database and the resulting *E*-value was taken as a measure of similarity between sequences (Atkinson et al., 2009). The network was visualized using Cytoscape (Shannon et al., 2003), where each sequence was represented as a node and edges were defined between any pair of nodes with an *E*-value of less than a threshold, using the Cytoscape force-directed layout. Similarly, a network was constructed to analyze protein sequences codified in the virion-sense strand. For this analysis, we retrieved all translated geminivirus sense genes (*V1*, *V2*, *V3*, and *V4*) and added the two movement protein genes from bipartite begomovirus (*BC1*-MP and *BV1*-NSP). The sequences of mastrevirus and begomovirus (2168 and 14136, respectively) were clustered to reduce the number of sequences and redundancy at 95 and 90% of identity, respectively. In total, the custom database comprises 1541 sequences belonging to the six proteins, including our query sequences.

### Phylogenetic and Recombination Analysis

To establish the phylogenetic relationships, we assembled three datasets. The first dataset included 66 nucleotide sequences of the full-length genome or DNA-A (in the case of begomoviruses) of representative species of all the genera of the *Geminiviridae* family obtained from a public database (NCBI). The two other datasets were amino acid sequences of the coat protein (CP) and the replication-associated protein (Rep) of the sequence of each species and the geminivirus representative species. All sequences in the datasets were aligned with MUSCLE (Edgar, 2004). For phylogenetic analysis, the nucleotide substitution model was chosen as the best-fitting model by using jModeltest v2.1.6 (Guindon and Gascuel, 2003; Darriba et al., 2012). The models of protein evolution were inferred with Prottest v3.4 (Guindon and Gascuel, 2003; Darriba et al., 2011). In both cases, we used the Akaike Information Criterion (AIC) to select the best-fitting evolution model. The phylogenetic reconstructions were performed by Maximun likelihood criterion using FastTree software for full-length sequences (Price et al., 2010) and PHYML 3.0 software for the CP and Rep datasets (Guindon et al., 2010) with 1000 bootstrap replicates. Tree topologies were observed with FigTree v1.4.1. Recombination analyses of full-length genomes (DNA-A for begomovirus included) were performed using RDP, GENECONV, MaxChi, Bootscan, 3Seq, Chimera and SiScan statistic methods implemented in the RDP4.67 software (Martin et al., 2015).

### CP Analyses for Prediction of the Tentative Type of Insect Vector

The same dataset of the CP amino acid sequences which was used for the phylogenetic tree was also used for the specificity-determining positions analyses (SDPs). SDPs are sites that show specific conservation patterns within subsets of proteins in a protein family alignment. Those sites are likely to be involved in the development of some kind of functional specificity. The goal of these analyses was the identification of amino acid residues that differ between groups of sequences, and to relate those amino acid residues to the viruses that have the same kind of insect vector. We hypothesize that those sites may be involved in the specificity of transmission; therefore, it is possible to predict the putative insect vector of this virus. SDPs were calculated with SPEER SERVER (Chakraborty et al., 2012) using MAFFT for sequence alignment (Katoh and Standley, 2013) and SCI-PHY for automated sub-grouping (Brown et al., 2007) setting the relative entropy term and the amino acids properties term weigh to one.

### Infectivity Assay in Tomato and Virus Detection

Infectivity assays were performed with RCA products of virus DNA genome by biolistic inoculation using a PDS-1000/He particle delivery system (Bio-Rad) in tomato plants. Plasmids containing cloned full-length DNA genome were digested with *Pst*I agarose-gel purification to obtain a monomeric component and then re-circularized by a ligation reaction (Knierim and Maiss, 2007; Lapidot et al., 2007). The RCA amplification product (5 μl) was precipitated on tungsten micro-particles and then inoculated into 10 Santa Clara tomato plants with 4–5 leaves. Plants were grown in greenhouse conditions with a 16/8 photoperiod.

In order to evaluate the virus infection, total DNA from apical leaves was purified 21 days after the inoculation and the viral genome was amplified by RCA and analyzed by *Sau3A*I restriction fragment length polymorphism (RFLP). Finally, we designed primers (NG_FW: 5′-ACTTCCAAAACTGGCTACAA-3′; NG_RV: 5′-AGAGCACATACCATCCAAAC-3′) to develop a PCR assay for specific virus identification. The reaction was as follows: 2 μl of total DNA, 0.25 mM each dNTPs, 2.5 mM of each primer and 1U of GoTaq DNA Polymerase (5 U/ml) (Promega, Madison, WI, United States). The temperature program used was: 94°C for 2 min, followed by 30 cycles of 94°C for 1 min, 51°C for 30 s, and 72°C for 2 min, with a final elongation step of 5 min at 72°C. The PCR product was evaluated by agarose gel electrophoresis and the presence of a 1375 bp DNA band confirmed the identity of the virus.

## Results

### Molecular Characterization

**FIGURE 1:**
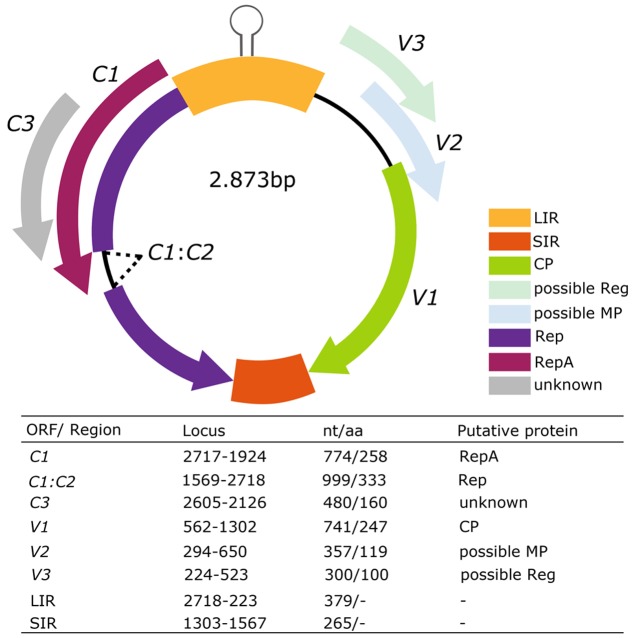
Genomic organization and ORFs sizes of the putative species tomato apical leaf curl virus (ToALCV). The table characterize of the open reading frames of ToALCV. Length (nt/aa), pairwise identity by the BLAST algorithm, protein motif identification by the Conserved Domain Tool (NCBI) and the SMART algorithm.

Tomato leaves with yellowing, reduction and wrinkling were collected in Yuto, Jujuy. We obtained a linear DNA fragment of about 2.8 kb generated from the digestion with *Pst*I of RCA product amplified from three symptomatic plants. These fragments were isolated, cloned and sequenced by primer walking. The full-length virus insert was about 2874 pb for the isolation [AR:Yuto:Tom419:2008]-MG491195, 2873 pb for the isolation [AR:Yuto:Tom420:2008]-MG491196 and [AR:Yuto:Tom424:2008]-MG491197. The BLAST algorithm identified *Alfalfa leaf curl virus* (ALCV) as the most related sequence in the GenBank database. There was 63% of full-length pairwise identity with ALCV, which is below the 78% of the species demarcation threshold proposed by ICTV for *Capulavirus* classification (Varsani et al., 2017). We found the conserved geminivirus nonanucleotide (TAATATTAC) in all three genomic sequences obtained. Therefore, we identified a new geminivirus and proposed to name it tomato apical leaf curl virus (ToALCV). Six ORFs that might codify to putative proteins were identified. The inferred genome organization has three virion-sense genes (*V1*, *V2*, and *V3*) and three complementary-sense genes (*C1*, *C1:C2* and *C3*) with two intergenic regions (LIR and SIR). The Replication-associated protein A (RepA) was codified by *C1* as it shares a conserved domain with the RepA of geminiviruses. *C1:C2* expressed for a spliced mRNA transcript and contained intron sequences. This protein shared sequence similarity with motifs conserved across the replication-associated protein (Rep) in geminiviruses, including the conserved motif I, II, and III, and the slightly different GRS motif (**Supplementary Figure S1**) (Nash et al., 2011). C3 predicted protein presented Rep-like motifs (*E* = 9.31e^-42^) in its sequence. On the other hand, the putative protein V1 was highly related with the geminivirus CP (*E* = 1.49e^-19^). V2 has a transmembrane domain which was detected by the SMART algorithm. V3 did not share any known conserved motif or domain with the database protein. In summary, the genome of ToALCV has six putative proteins and two intergenic regions (**Figure 1**).

### Sequence Similarity Network Analysis

**FIGURE 2:**
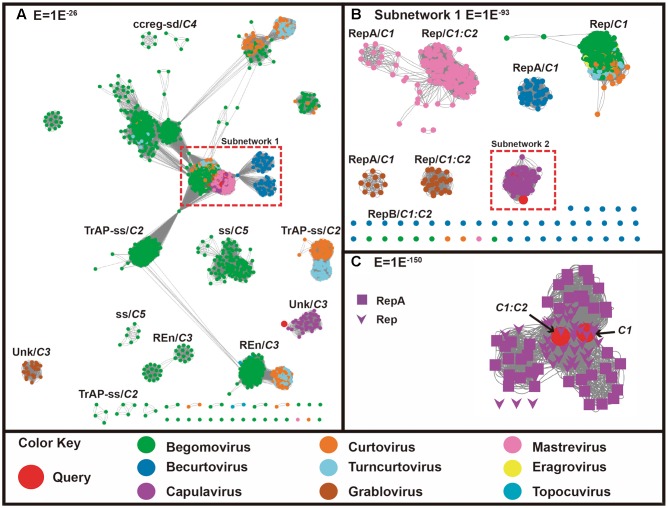
Sequence similarity networks (SSNs) including 2910 geminiviral complementary-sense coded protein sequences (codified by *C1*, *C1:C2*, *C2*, *C3*, *C4*, and *C5* genes). Sequences are represented as nodes and the pairwise relationships, as edges (lines) between nodes. Nodes are colored according to the virus genera to which they belong, while the query sequences are colored in red. **(A)** SSNs using a permissive threshold (*E*-value = 1E^-26^). When sequences break out into clusters where the annotation is coincident, the name of the protein/function is indicated. One of the query sequences (putative C3) (red circle) clusters with the C3 sequences of capulavirus. Dotted red boxes enclose a group of sequences that was chosen for further analysis using more stringent thresholds (nodes are associated with more significant relationships). **(B)** Subnetwork 1, composed by 905 sequences, is shown at *E*-value threshold = 1E^-93^. The two query sequences cluster with capulavirus (enclosed in red dotted line). The subnetwork is further analyzed in **(C)** Subnetwork 2, which includes 91 sequences, and is shown at a more stringent threshold (*E*-value = 1E^-150^). Here the shape of the node indicates the protein annotation/function. The query sequences are closely related to the Rep associated protein of capulaviruses.

The complementary sense translated protein similarity network analysis indicates that the proteins coded by *C1* and *C1:C2* of our query genes clustered with all geminiviral RepA, RepB and Rep proteins (**Figures 2A,B**). **Figure 2B** shows further decomposition of a subnet (subnetwork 1) in the more stringent threshold value of 1E^-93^. It can be observed that, using this threshold, the Rep and RepA of mastrevirus, becurtovirus, grablovirus are clearly separated into different similarity groups, while the Rep and RepA of capulavirus lay in one group. The putative RepA-*C1* and Rep-*C1:C2* of ToALCV grouped with capulavirus. In subnetwork 2, with a more restrictive threshold of 1E^-150^, the RepA-*C1* and the Rep-*C1:C2* of ToALCV remained attached to the capulavirus Rep/RepA group, although both proteins seem to be closely related to the capulavirus Rep (**Figure 2C**). Interestingly, all the rest of the geminivirus genera that codified only a Rep stayed in the same group. Meanwhile, C3 protein of ToALCV grouped with C3 proteins of capulaviruses forming a detached group from the core with unknown function (**Figure 2B**).

**FIGURE 3:**
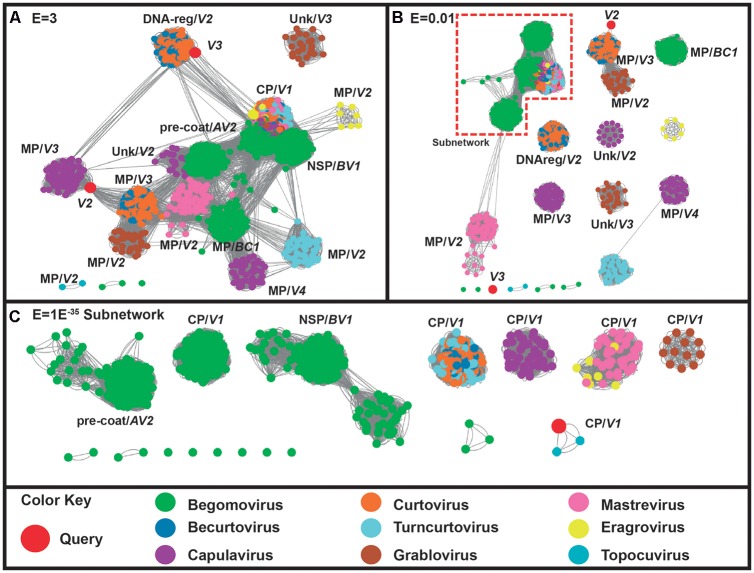
Sequence similarity networks (SSNs) including 1541 sense coded protein geminiviral sequences (codified by *V1*, *V2*, *V3*, *V4*, *BC1*-MP and *BV1*-NSP genes). Sequences are represented as different colored nodes and the pairwise relationships, as edges (lines) between nodes. The nodes are colored according to the virus genera to which the sequence belongs, while the query sequences are colored in red. **(A)** SSNs using a permissive threshold (*E*-value = 3). When sequences break out into clusters where the annotation is coincident, the name of the coded protein is indicated. **(B)** The network is shown at *E*-value threshold = 0.03. **(C)** The subnetwork that comprises 898 sequences at an *E*-value threshold = E^-35^.

On the other hand, the proteins codified by virion-sense translated genes (*V1*, *V2*, and *V3*) of ToALCV clustered into a group that does not include the proteins of capulavirus. *V1* is clearly related to the of all the geminiviruses at high threshold values (*E* = 3) (**Figure 3A**). Finally, the subnetwork in a threshold cut of *E* = 1E^-35^ presented different CP-*V1* groups for capulaviruses, grabloviruses and begomoviruses (**Figure 3C**). The CPs of curtoviruses, becurtoviruses and turncurtoviruses are closely related and they formed another group. The same happened with the CPs of mastreviruses and eragroviruses. Contrary, the CP-*V1* of ToALCV sequences clustered with the unique CP of topocuviruses and not with the CPs of capulaviruses. V2 grouped with movement proteins (MP) allowing the deduction of its function and suggesting a relationship with the MPs of curtoviruses and becurtoviruses (**Figures 3A,B**). V3 grouped with the proteins of curtoviruses and becurtoviruses involved in the regulation of the relative level of the ssDNA and dsDNA (Reg protein) (**Figure 3A**). However, V3 query protein was separated from all the other proteins at *E* = 0.03 threshold value making it difficult to confirm this proposed function (**Figure 3B**).

According to the protein similarity of the sequences and the order of codification, ToALCV presents modular organization. The complementary-sense gene products (RepA, Rep and C3) are similar to those of capulaviruses while the viron-sense gene products (CP, MP and V3) are similar to those belonging to topocuviruses, curtoviruses and becurtoviruses.

### Phylogenetic Relationships

**FIGURE 4:**
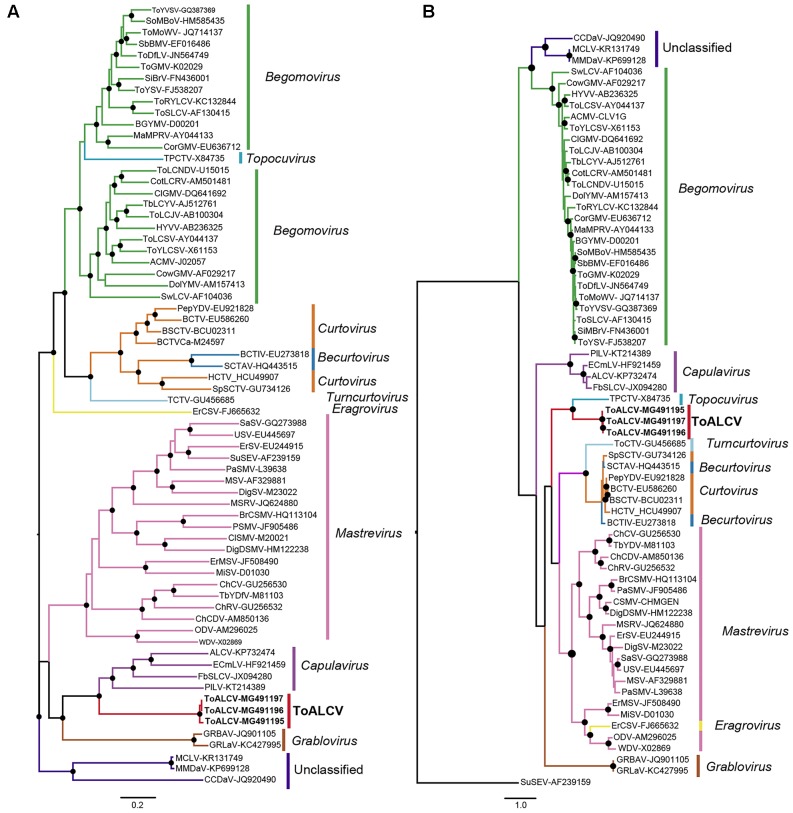
**(A)** Maximum likelihood phylogenetic tree of 66 full length genomes or DNA-A (bipartite begomovirus). **(B)** Maximum likelihood phylogenetic tree obtained with amino acid sequence alignments of the CP of the same 66 geminiviruses. Filled circles indicate 70 or more bootstrap percentages.

The evolutionary relationship between ToALCV and other known geminiviruses was inferred using full-length genome nucleotide sequences and amino acid sequences of the CP and the Rep proteins. The molecular evolutionary models were LG+I+G+F and VT+I+G for the CP and the Rep alignments, respectively. The phylogenetic tree for the full-length genome revealed that *Capulavirus* was the closest related genera. The three isolates ([AR:Yuto:Tom419:2008]-MG491195, [AR:Yuto:Tom420:2008]-MG491196 and [AR:Yuto:Tom424:2008]-MG491197) formed a monophyletic group with ALCV, EcmLV, FbSLCV and PLLV (**Figure 4A**). Nevertheless, ToALCV shaped a monophyletic group with the topocuvirus *Tomato pseudo-curly top virus* (ToPCTV) in the CP- phylogenetic tree (**Figure 4B**), while it grouped with the sequences of the capulavirus in the Rep-tree (**Supplementary Figure S2**), which corresponds with the results showed in the full-genome tree (**Figure 4A**).

### Recombination Analysis

**FIGURE 5:**
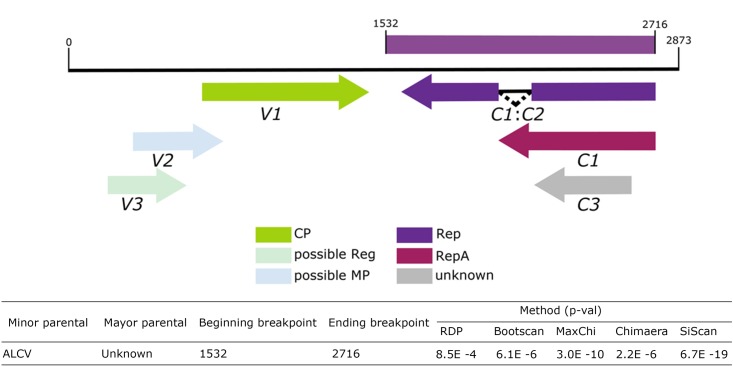
Schematic representation of the recombination event detected by RDP4.67 in the ToALCV genome and table with corresponding the *p*-values.

Five statistical methods, RDP (*p*-value = 8.5E^-4^), Bootscan (*p*-value = 6.1E^-6^), MaxChi (*p*-value = 3.0E^-10^), Chimera (*p*-value = 2.2E^-6^) and SiScan (*p*-value = 6.7E^-19^) in RDP v4.67 identified evidence of recombination for ToALCV. These analyses showed that the sequences of ALCV in the analyzed dataset are the sequences that most closely resemble the parental sequence; however, the major parental sequence remains unknown. The beginning breakpoint is at nucleotide 1532 and the ending breakpoint is at nucleotide 2716 involving a segment of the putative Rep sequences of ToALCV (**Figure 5**).

### Vector Specificity Prediction Based on Capsid Protein Amino Acidic Sequences

The automatic subgrouping defined nine subgroups that include geminiviruses that share the same type of insect vector. In that way, the subgroups 1, 2, and 3 include geminiviruses transmitted by the leafhopper (*Cicadellidae*). The subgroups 1 and 4, geminiviruses transmitted by the treehopper (*Membracidae*). The subgroup 8 contains those transmitted by the aphid (*Aphididae*) and subgroup 9 includes begomoviruses transmitted by the whitefly (*Aleyrodidae*). Interestingly, the subgroup 7 contains geminiviruses that have not been assigned to a family because their insect vector has not been identified. The geminiviruses transmitted by the leafhopper are divided into three subgroups and the geminiviruses transmitted by the treehopper, in two, according to the species of the insect-vector. SDP analyses clustered the three sequences of ToALCV with ToPCTV_X84735 for which the transmission vector is *Micrutalis malleifera*. Therefore, we propose that a treehopper (*Membracidae*) could be the vector of this virus. Also, a set of nine amino acid residues (L68, E86, A103, W112, Y203, N206, I212, I215 and P226) with the highest scoring positions was predicted for the CP that could determine differences in virus transmission specificity (**Supplementary Figure S3**). The predicted amino acid residues with functional specificity were concentrated in two positions in the CP; one, in the last part of the amino-terminal region, and the other, in the carboxy-terminal part.

### Infectivity and Symptoms Development by Genomic Biolistic Inoculation

**FIGURE 6:**
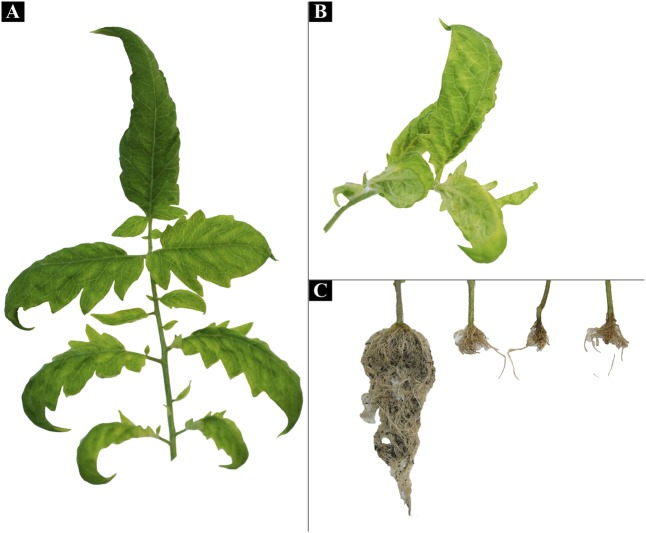
Biolistic infected plants symptoms. **(A,B)** Leaf interveinal yellowing and curling symptoms exhibited by ToALCV of inoculated plants with [AR: Yuto: Tom424:2008] RCA product by the biolistic method. **(C)** Root hypotrophy in plants infected with [AR: Yuto: Tom424:2008].

A total of 10 plants were biolistic inoculated with the RCA products of previously cloned full-length [AR:Yuto:Tom419:2008] and [AR:Yuto:Tom424:2008] genomes. Three plants showed interveinal yellowing, apical leaf curling and extreme root hypotrophy (**Figure 6**). The evaluation of the presence of ToALCV in the symptomatic plants by PCR with the specific primers resulted in the amplification of a 1400 bp fragment which confirmed the infection. Moreover, the *Sau3A*I RCA-RFLP (1027, 923, 735, and 188 bp) patterns obtained from the infected plants were the same as the predicted *in silico* patterns for ToALCV.

## Discussion

We identified and characterized a novel monopartite geminivirus infecting tomato in Argentina. The name tomato apical leaf curl virus (ToALCV) is proposed. A BLAST comparison shows that the nucleotide sequence only aligns in a small portion of this geminivirus genome, which matches the Rep portion of ALCV and that there is only 63% of nucleotide identity in the full-length genome. This indicates that it is highly divergent from all the *Geminiviridae* family. The full-length genome phylogenetic tree of the three isolates of ToALCV revealed that they cluster within capulavirus clades and share a common ancestor (**Figure 4A**). The same relationship was obtained with the Rep protein phylogenetic analyses (**Supplementary Figure S1**), but not with the CP protein (**Figure 2B**). In this case, the three isolate amino acid sequences formed a monophyletic group with ToPCTV, a topocuvirus that has not been identified in South America so far (Briddon et al., 1996). This lack of phylogenic relationship agreement of the Rep and the CP of ToALCV contrasts with the coincident results obtained for capulaviruses, where all proposed species clustered together in the capulavirus group in both trees (Varsani et al., 2017). The low percentage of nucleotide identity of the full-length genome, the different evolutionary relationships of the Rep and CP proteins, in addition to the recombination evidence, support a modular organization and a different evolutionary history of the virion-sense and complementary-sense frame of the ToALCV genome. A strong signal of recombination was found in the Rep segment of this virus. Beginning and ending recombination breakpoints are in nucleotides 1532 (within the small intergenic region, SIR) and 2716 (within the long intergenic region, LIR), respectively. The sequences in the recombination assay dataset that most resemble the recombination parental for the Rep segment belong to ALCV, agreeing with the results in the phylogenetic analysis. No other parental could be determined, which may be because the origin of this new species is ancient or its parental sequences have not been described yet. Topocuviruses and curtoviruses are examples of modular genome arrangement that is thought to have arisen through the recombination of fragments belonging to different Geminivirus genera (Stanley et al., 1986; Briddon et al., 1996; Klute et al., 1996; Varsani et al., 2009). The nucleotide sequence identity of virion-sense genes of becurtoviruses like *Beet curly top Iran virus* (BCTIV) are mostly related to those of curtoviruses, whereas the complementary-sense genes are distally related to mastreviruses (Yazdi et al., 2008; Bozorgi et al., 2017). Today, it is a challenge to determine with certainty, which genera are recombinant and which are parental; as with BCTIV, which was proposed to be involved as parent in the inter-genus recombination that originated curtoviruses (Varsani et al., 2009). The three replication modes postulated for geminiviruses; complementary strand replication (CSR), rolling circle replication (RCR) and recombination dependent recombination (RDR) have the potential to induce recombination in different parts of the genome. This plays a crucial role in geminivirus evolution as a motor for the switching of the host and the emergence of viruses (Saunders et al., 1991; Jeske et al., 2001; Preiss and Jeske, 2003; Lefeuvre and Moriones, 2015; Richter et al., 2016). When genetically distinct ssDNA virus genomes co-replicate within the same nucleus, there are a number of different mechanistic and selective processes that could determine the patterns of recombination that are conserved (Martin et al., 2011). The v-ori recombination hot spot was mapped for several geminiviruses (Lefeuvre et al., 2009). Complementary-sense genes in the geminivirus experience increased their recombination rate in relation to virion-sense genes, possibly due to mechanistic interferences between the transcription and replication complexes during RCR (Lefeuvre et al., 2009).

Genome organization of ToALCV showed the arrangement of six predicted ORFs encoding three viral-sense genes (*C1*, *C1:C2*, *C3*), three complementary-sense genes (*V1*, *V2*, *V3*) and two intergenic regions, LIR and SIR (**Figure 1**). Other geminivirus genera with two intergenic regions in their genome are becurtovirus, capulavirus, grablovirus, eragrovirus, mastevirus and CCDaV (Varsani et al., 2014, 2017). ToALCV has two putative genes involved in the replication by RCA: *C1*, which codified for a RepA protein, and *C1:C2*, which codified for a Rep fusion protein in which a splicing processing step was identified. Similar splicing of *C1:C2* was described for mastreviruses, becurtoviruses, capulaviruses, grabloviruses and one begomovirus (*Mungbean yellow mosaic virus*) (Mullineaux et al., 1990; Dekker et al., 1991; Boulton, 2002; Shivaprasad et al., 2005; Bozorgi et al., 2017). The complementary-sense codified proteins are clearly similar to capulavirus (**Figure 2**). It is important to point out that the predicted expression products of *C1* (RepA) and *C1:C2* (Rep) presented more similarities with *C1:C2* (Rep) of the capulavirus species (**Figure 2C**). As with capulavirus and grablovirus, they have a large C3 that overlaps with the Rep sequences (**Figure 1**). The C3 of ToALCV also has some degree of protein sequence similarity with capulaviruses, although no function is postulated yet for any of these proteins (**Figures 2B,C**). Of the virion-sense coding region, the main protein amongst all geminiviruses is the CP, due to its features to be conserved. The *V1* gene codified for a putative CP that shares significant amino acid sequence similarity with the CP of ToPCTV. In addition, the evolutionary relationships with topocuviruses were displayed in the CP amino acid phylogenetic tree (**Figures 1**, **3C**, **4B**). Although the CP of ToALCV is clearly very similar to topocuvirus, it is not obviously derived from topocuvirus through recombination because no recombinant event was identified in these fragments in the analyses. The localization of the gene *V2* that codified for a putative MP is immediately upstream of the CP like the other members of the *Geminiviridae* family and has sequence similarity with the MP proteins of curtoviruses and becurtoviruses (**Figures 1**, **3**). *V3* gene of ToALCV codified for the most divergent protein, but in the SSN analyses, (*E* = 3) V3 grouped with the Reg protein of curtoviruses and becurtoviruses (**Figures 3A,B**).

It is known that changes in the amino acidic sequence of the CP in geminiviruses can modify the vector specificity (Briddon et al., 1990). Based on that, we performed an SDP analysis to see if the CP amino acidic sequences of geminiviruses with similar insect vector exhibited features more akin amongst them. We found that a clustering based only on the amino acidic sequences (with no other information added) rendered 8 subgroups of proteins. Proteins in those subgroups surprisingly belong to viruses that use the same vector for its transmission. The nine highest scoring SDP amino acid positions are enough to separate the geminiviruses according to their specific transmission vector (**Supplementary Figure S3**). Our sequences were clustered with topocuviruses whose vector is the treehopper, *Micrutalis malleifera* (Briddon et al., 1996). This result sustains the prediction that the insect vector of ToALCV is probably the treehopper. Transmission assays need to be made in order to confirm this prediction. In spite of this, we have no record of any increase in the treehopper population or any new treehopper species threatening tomato cultivated in the northwest region of Argentina when these viruses were isolated. There is a report of *Micrutalis malleifera* Fowler in our country (Christensen, 1943), but its presence does not necessarily make it its vector and, besides, there are other reported treehoppers in Argentina (de Remes-Lenicov, 2014).

The SDP analysis of the CP was carried out, apart from the phylogenetic analyses of the CP here presented, to contribute with the prediction of the type of insect that could transmit ToALCV. Thus, the whitefly, the aphid, the treehopper and the leafhopper subgroups were defined and ToALCV was clustered within the treehopper subgroup (**Supplementary Figure S3**). The SDP analysis was useful to track down the crucial amino acid residue that could be involved in insect transmission specificity of geminiviruses (**Supplementary Figure S2**). The predicted amino acid residues with functional specificity were concentrated in two positions in the CP: one, in the last part of the amino-terminal region, and the other, in the carboxy-terminal part (**Supplementary Figure S3**). The CP is a multifunctional protein, so it was associated with virus genome packaging, movement and viral DNA replication, in addition to insect transmission (Fondong, 2013). There are reports that define amino acid residues and protein domain involved in whitefly transmission which have been mapped to the central part of the CP (Noris et al., 1998; Kheyr-Pour et al., 2000; Höhnle et al., 2001; Liu et al., 2001; Unseld et al., 2001). In addition, an N-terminal nuclear localization signal was found in the CP that interacts with the GroEL produced by the whitefly *Bemisia tabaci* endosymbiotic bacteria, probably to protect the virions in the haemolymph of the insect vector (Kunik et al., 1998; Morin et al., 2000; Yaakov et al., 2011; Rana et al., 2012). The amino acid residue predicted by SDP analysis could guide the design of experiments to define motifs of the CP involved in vector transmission of geminiviruses.

The biolistic inoculation with ToALCV infected tomato plants and developed characteristic viral symptoms including internerval yellowing and apical leaf curling (**Figure 6**). Furthermore, the three infected plants presented a marked root hypotrophy. This root symptom is rare in viral infections, but some authors describe similar behaviors within the *Geminiviridae* family, specifically in begomoviruses that infect sweet potato and cassava (Owor et al., 2004; Villordon and Clark, 2014).

ToALCV could be assigned to a new *Geminivirus* genus, with the tentative name of tomapivirus, due to its unique genome organization, its recombination origin and the evolutionary relationships of the nucleotide of the full-length and CP sequences. The unique modular genome arrangement of ToALCV has a capulavirus-like Rep, RepA and C3, a topocuvirus-like CP and a curtovirus and becurtoviru*s*-like *V2*-MP and V3 proteins. Using SSN to analyze the relationships of sequences and function among proteins codified by the different genus of the *Geminiviridae* family supported the taxonomy proposal for ToALCV. Moreover, the identification of amino acid residues of the CP was employed to predict that a treehopper could be the vector of this virus but transmission assays need to be performed to confirm this.

## Author Contributions

CVM, VB, CF, and PLL conducted the experiment. CVM, ET, CM-B and PLL analyzed the data and wrote the manuscript. All authors read and approved the final manuscript.

## Conflict of Interest Statement

The authors declare that the research was conducted in the absence of any commercial or financial relationships that could be construed as a potential conflict of interest.

## Footnotes

1 http://www.geneious.com

2 http://blast.ncbi.nlm.nih.gov/

## Supplementary Material

**FIGURE S1 |** Alignment of the Rep 5′ ends of ALCV and the sequences of the three ToALCV isolations. Motif I, Motif II, Motif III and the GRS region are indicated with boxes.

**FIGURE S2 |** Maximum likelihood phylogenetic tree obtained with amino acid sequence alignments of the Rep of 66 geminiviruses. Filled circles indicate 70 or more bootstrap percentages.

**FIGURE S3 |** Specificity-determining positions alignment for the CP amino acid sequences of the *Geminiviridae* family. **(Top)** CP multiple sequence alignment colored by clustalX code. The sequences were automatically divided into 9 groups by SCI-PHY and SDPs calculated by the SPEER server. Colored boxes on the left indicate the different genus of the *Geminiviridae* family. The names of the sequences indicate the acronym, accession number, known insect vector and subgroup clustering. The numbered boxes on the center indicate the different clusters formed in the analysis. Subgroup 5 (where the interest sequences are) is pushed on top for clarity. The arrows show the highest scored amino acids that are informative to the clustering. **(Bottom)** Histograms of the conservation and quality of the alignment colored from brown to yellow (from lower to higher conservation and quality, respectively). The last histogram shows the sequence logo.
