# Supplementary figures and images for: Tomato Apical Leaf Curl Virus: A Novel, Monopartite Geminivirus Detected in Tomatoes in Argentina
Source: Front Microbiol. 2018 Jan 12;8:2665. doi: 10.3389/fmicb.2017.02665 (PMC5770407; doi:10.3389/fmicb.2017.02665)

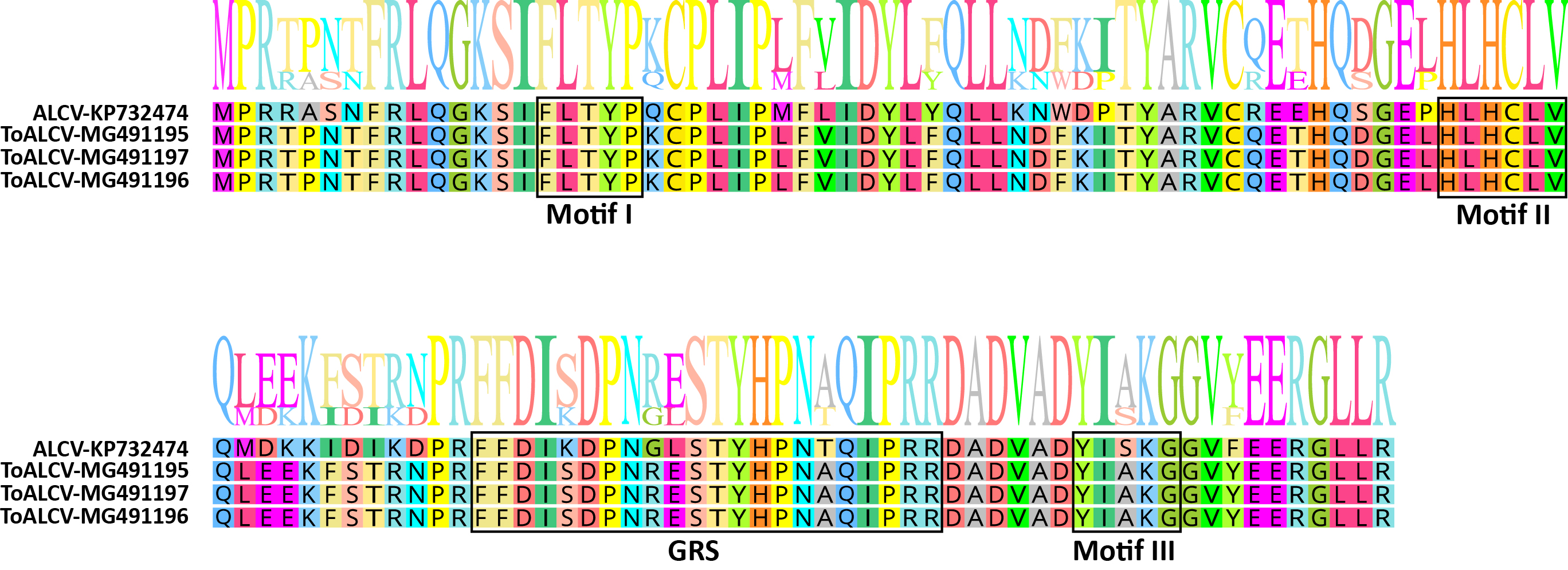

Supplement: Supplementary file 1 [file Image_1.JPEG]

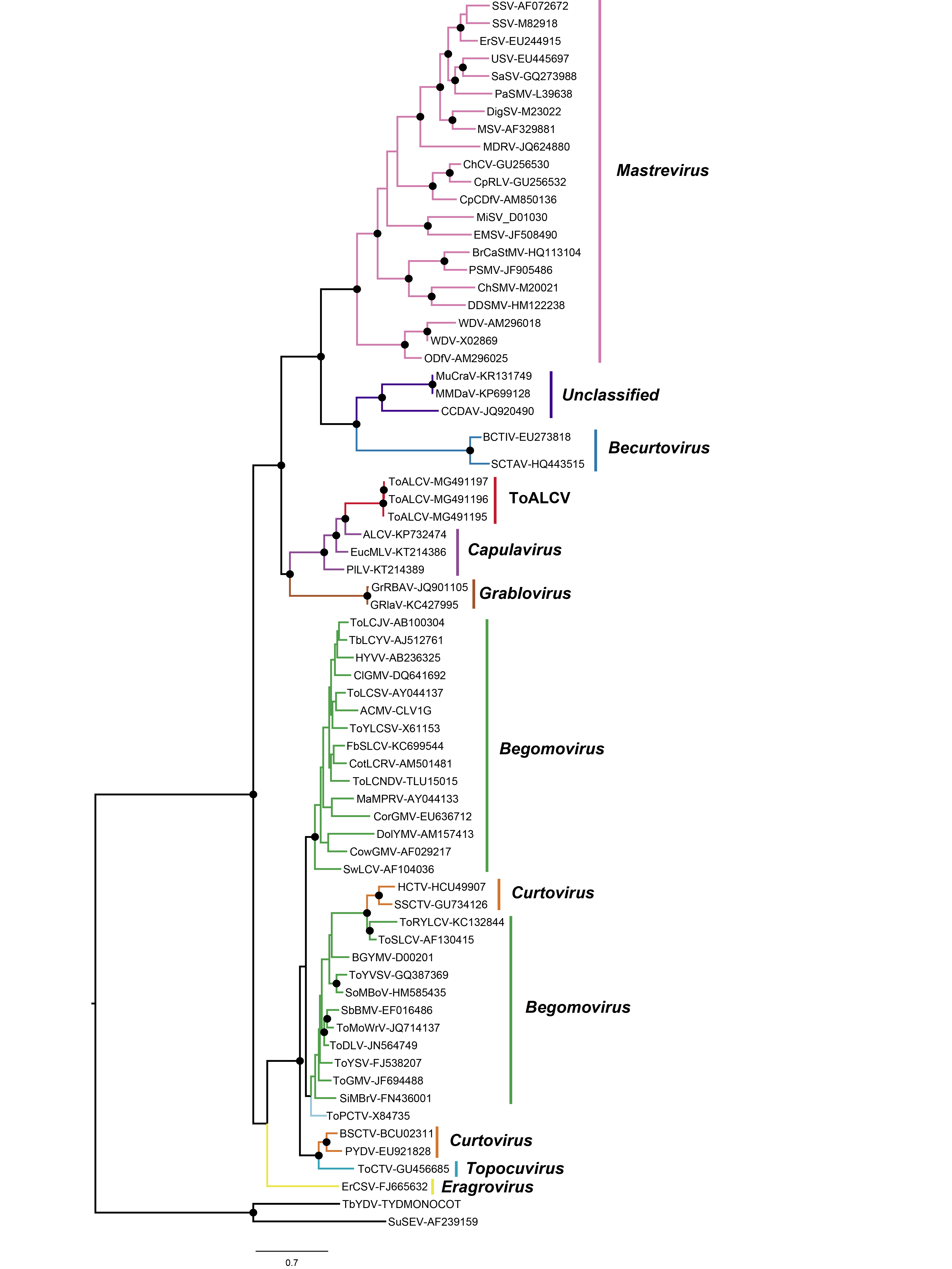

Supplement: Supplementary file 2 [file Image_2.JPEG]

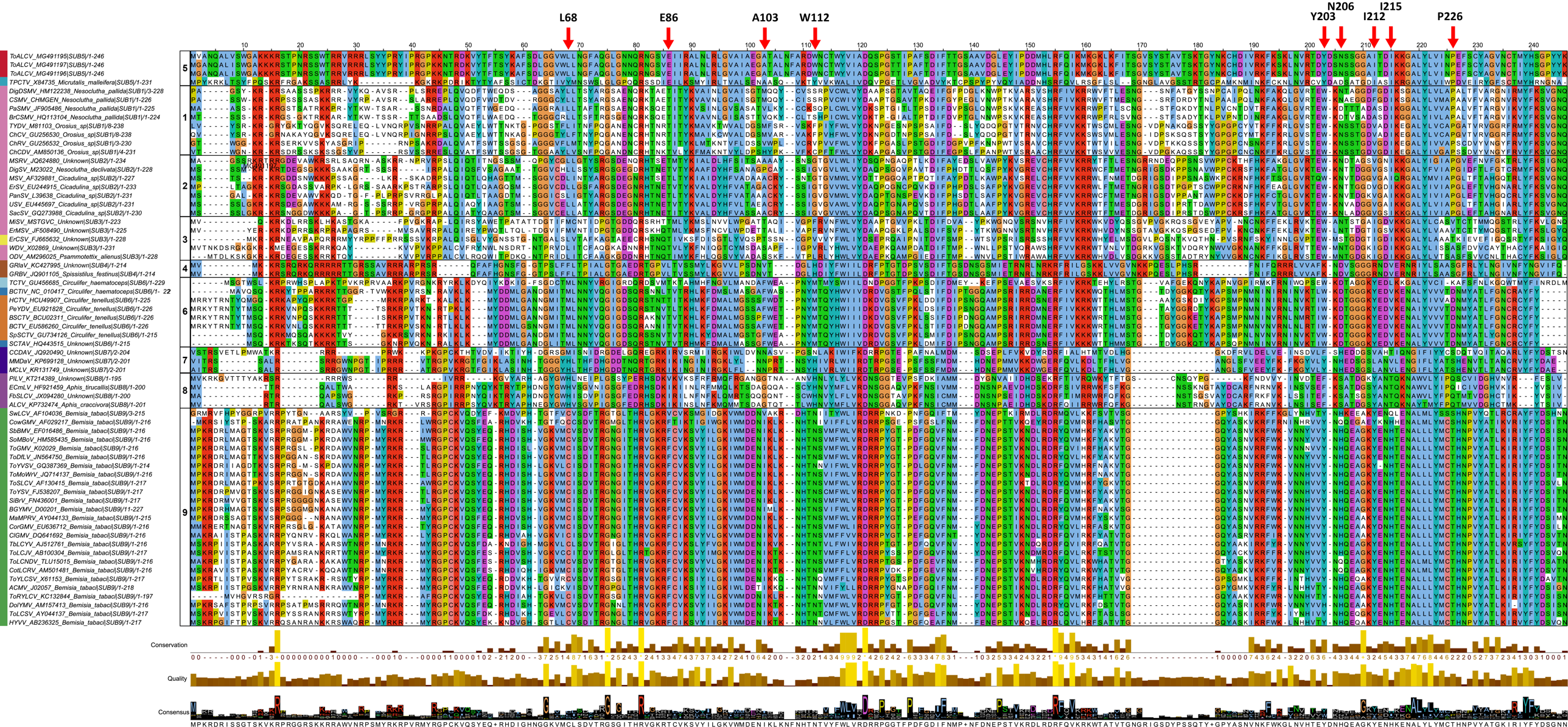

Supplement: Supplementary file 3 [file Image_3.JPEG]
